# Neoadjuvant chemotherapy versus neoadjuvant chemoradiotherapy for cancer of the esophagus or the gastroesophageal junction: A meta-analysis based on clinical trials

**DOI:** 10.1371/journal.pone.0202185

**Published:** 2018-08-23

**Authors:** Xin Zhao, Yiming Ren, Yong Hu, Naiqiang Cui, Ximo Wang, Yunfeng Cui

**Affiliations:** 1 Tianjin Medical University, Tianjin, China; 2 Department of Surgery, Tianjin Nankai Hospital, Nankai Clinical School, Tianjin Medical University, Tianjin, China; 3 Department of Bone and Joint, Tianjin Union Medicine Center, Tianjin, PR China; Duke Cancer Institute, UNITED STATES

## Abstract

**Background:**

The benefit of neoadjuvant chemotherapy and neoadjuvant chemoradiotherapy for treating cancer of the esophagus or the gastroesophageal junction remains controversial. In the present study, we conducted a comprehensive meta-analysis to examine the efficacy of these two management strategies.

**Methods:**

The MEDLINE (PubMed), SinoMed, Embase, and Cochrane Library databases were searched for eligible studies. We searched for the most relevant studies published until the end of September 2017. Data were extracted independently and were analyzed using RevMan statistical software version 5.3 (Cochrane Collaboration, http://tech.cochrane.org/revman/download). Weighted mean differences, risk ratios (RRs), and 95% confidence intervals (CIs) were calculated. Cochrane Collaboration’s risk of bias tool was used to assess the risk of bias. In this comprehensive meta-analysis, we examined the efficiency of neoadjuvant chemotherapy and neoadjuvant chemoradiotherapy for the treatment of cancer of the esophagus or the gastroesophageal junction as reported in qualified clinical trials.

**Results:**

Six qualified articles that included a total of 866 patients were identified. The meta-analysis showed that for 3-year and 5-year survival rates in primary outcomes, the results favored neoadjuvant chemoradiotherapy strategies compared with neoadjuvant chemotherapy (RR = 0.78, 95% CI = 0.62–0.98, P = 0.03; RR = 0.69, 95% CI = 0.50–0.96, P = 0.03, respectively). In terms of secondary outcomes, neoadjuvant chemoradiotherapy significantly increased the rate of R0 resection and pathological complete response as well (RR = 0.87, 95% CI = 0.81–0.92, P < 0.0001; RR = 0.16, 95% CI = 0.09–0.28, P < 0.00001, respectively). However, there were no significant differences in postoperative mortality between the two groups (RR = 1.85, 95% CI = 0.93–3.65, P = 0.08). For the results of postoperative complications, revealed that there was a statistically significant difference between the two groups in the incidence of postoperative complications such as pulmonary, anastomotic leak and cardiovascular complications. The subgroup analysis of patients with esophageal adenocarcinoma or squamous cell carcinoma showed that both esophageal adenocarcinoma and squamous cell carcinoma patients achieved a high rate of R0 resection (RR = 0.85, 95% CI = 0.77–0.93, P = 0.0006; RR = 0.88, 95% CI = 0.81–0.96, P = 0.005, respectively) and pathological complete response benefit of neoadjuvant chemoradiotherapy (RR = 0.23, 95% CI = 0.09–0.57, P = 0.001; RR = 0.18, 95% CI = 0.03–0.96, P = 0.05, respectively).

**Conclusion:**

Our findings suggested that compared with neoadjuvant chemotherapy, neoadjuvant chemoradiotherapy should be recommended with a significant long-term survival benefit in patients with cancer of the esophagus or the gastroesophageal junction. In view of the clinical heterogeneity, whether these conclusions are broadly applicable should be further determined.

## Introduction

Esophageal cancer has the sixth highest mortality rate of malignant tumors worldwide, with more than 400,000 related deaths annually [1]. Histologically, esophageal cancer comprises mainly squamous cell carcinoma and esophageal adenocarcinoma. A recent epidemiological study showed that up to 2015, the incidence of esophageal cancer ranked 8^th^ in all malignant tumors, and mortality rates were the fourth highest [2]. Currently, surgical treatment is the main method for the treatment of esophageal cancer, but the effectiveness of surgery alone has been unsatisfactory, and the median survival of patients has rarely exceeded eighteen months; thus, surgery only leads to relatively few long-term survivors [3,4]. Despite advances in treatment, the prognosis of esophageal cancer still remains unoptimistic, with the rate of 5-year survival less than 20% [5]. In the past 20 years, neoadjuvant chemotherapy for resectable esophageal carcinoma has been a research area of great activity.

Neoadjuvant therapy has gradually become consummate, including neoadjuvant chemotherapy and neoadjuvant chemoradiotherapy, which have convincingly shown benefit to patients with cancer of the esophagus or the gastroesophageal junction [6,7]. Although some advances in the treatment of esophageal cancer have occurred, overall survival remains poor [8]. Worldwide, in several randomized trials comparing neoadjuvant chemotherapy to surgery alone, the superiority of neoadjuvant chemotherapy in long-term survival was demonstrated [9,10]. In the following studies, neoadjuvant chemoradiotherapy has received much attention in the treatment of this carcinoma. A recent meta-analysis showed that neoadjuvant chemoradiotherapy was associated with the improvement of the long-term survival rate as well [11]. Both clinical trials and meta-analyses have reported that neoadjuvant therapy is more beneficial for patients with esophageal or gastroesophageal junction cancer [12–14]. However, it remains speculative whether neoadjuvant chemoradiotherapy is superior. The latest comprehensive systematic review and meta-analysis of the options for neoadjuvant therapy in the treatment of esophageal cancer showed that neoadjuvant chemoradiotherapy should be the standard preoperative treatment strategy for locally advanced esophageal squamous cell carcinoma; for adenocarcinoma, neoadjuvant chemotherapy alone may be the best choice to avoid the risk of complications of radiotherapy [15]. Another published study that compared neoadjuvant chemoradiotherapy with neoadjuvant chemotherapy concluded that neoadjuvant chemoradiotherapy can achieve a long-term survival benefit [16]. Therefore, it is difficult to identify whether neoadjuvant chemoradiotherapy has a clear advantage compared with neoadjuvant chemotherapy because of the limited studies; additionally, the sample size should be examined. Herein, we perform an updated meta-analysis of available data to determine if neoadjuvant chemoradiotherapy is superior to neoadjuvant chemotherapy in patients with cancer of the esophagus or the gastroesophageal junction.

## Materials and methods

This meta-analysis was performed under the recommendations of preferred reporting items for systematic reviews and meta-analyses (PRISMA) guidelines [17].

### Literature search strategy and data collection

A computerized search was conducted using MEDLINE (PubMed), SinoMed, Embase, and Cochrane Library databases. We searched for the most relevant randomized controlled trials (RCTs) published up to the end of September 2017, using combinations of the following keywords: “esophageal or oesophageal or gastro-oesophageal junction” AND “cancer or carcinoma or neoplasm” AND “neoadjuvant or preoperative” AND “chemoradiotherapy or radiotherapy or radiation” AND “chemotherapy” AND “clinical trial”. All reference lists from the trials selected by electronic searching were scanned to identify other relevant trials. The search was limited to human subjects and English language published studies.

### Eligibility and exclusion criteria

The inclusion criteria were the following: (1) the study included outpatients of either sex, aged 18 to 70 years, with a clinical, endoscopic, and histological diagnosis of esophagus or gastroesophageal junction carcinoma; (2) RCTs that compared neoadjuvant chemotherapy with neoadjuvant chemoradiotherapy for treating esophagus or gastroesophageal junction carcinoma (either esophageal adenocarcinoma or squamous cell carcinoma); (3) the rate of R0 resection, pathological complete response, and the survival rate were demonstrated in the study; and (4) studies covering the same populations were represented by only the most eligible study.

The exclusion criteria were as follows: (1) data description or sample information that was insufficiently clear; (2) no comparison between neoadjuvant chemotherapy and neoadjuvant chemoradiotherapy was made; and (3) case reports, abstracts, conference reports, reviews and reports of other experiments.

### Assessment of quality and risk of bias

Two reviewers independently extracted and checked the research data to ensure consistency. The quality of trials which were designed with control and treatment groups was assessed using Review Manager (Version 5.3; The Cochrane Collaboration, Oxford, UK). The risks of bias for RCT studies were evaluated with the Cochrane Collaboration’s risk of bias tool. Seven parameters were used to evaluate the quality of each included study: random sequence generation, allocation concealment, blinding, incomplete outcome data, selective outcome reporting, and other risks. Items were judged as "low risk", "unclear risk", or "high risk". Advice was sought through discussion or from a third partner to resolve inconsistent evaluations.

### Outcome indices of literature

Primary outcome measures included 3-year and 5-year survival rates. The rate of R0 resection, pathological complete response, perioperative mortality, postoperative complication and hospital stay were defined as secondary outcomes.

### Statistical analysis

In the systematic review, the meta-analysis was performed using the software Review Manager 5.3 (Cochrane Collaboration, http://tech.cochrane.org/revman/download). For dichotomous outcomes of the extracted data, risk ratios (RRs) and 95% confidence intervals (CI) were calculated; for continuous outcomes, weighted mean differences and 95% CI were used. Heterogeneity was assessed using the Q test and I^2^ statistic [18]. Statistical significance was set at P < 0.05. If there was significant heterogeneity (P ≦ 0.05, I^2^ ≧ 50%), a random-effects model was adopted; otherwise, fixed-effects models were applied if there was no significant heterogeneity (P ≧ 0.05, I^2^ ≦ 50%) [19]. When the interquartile range (IQR) and median were given instead of standard deviations (SD), we converted the data using Hozo’s algorithm to estimate the standard deviation [20].

We also performed a sensitivity analysis to assess the stability of the results and investigate the influence of each study by omitting a single study sequentially. Publication bias was shown by a funnel plot.

## Results

### Data extraction

**Fig 1:**
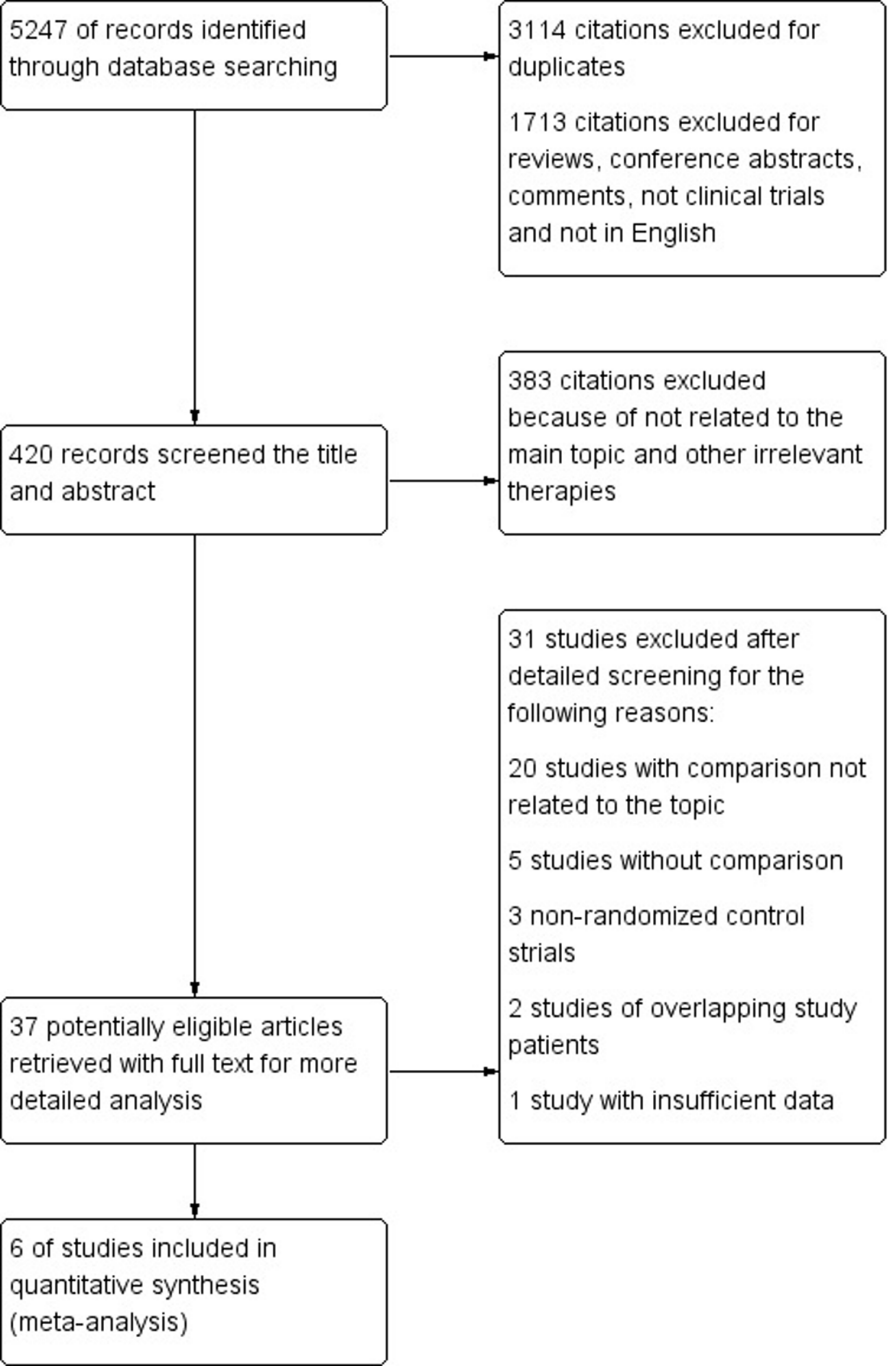
Flow diagram for selection of clinical trials included in the meta-analysis.

Of the 5,247 citations identified based on a study of the subject and summary of the literature, 3,114 were excluded due to duplication and 2,096 citations were excluded for obviously irrelevant records. Thirty-seven full-text studies were evaluated for further assessment; finally, six studies that met our inclusion criteria were identified. A detailed study flow diagram is shown in Fig 1.

### Study characteristics of included studies

In accordance with the search strategy and study selection criteria, six trials were identified for inclusion in this meta-analysis [21–26]. The characteristics of the included studies are presented in Table 1. The total number of patients within these six trials was 866. Across these studies, a total of 431 patients were treated with neoadjuvant chemotherapy and 435 patients were treated with neoadjuvant chemoradiotherapy. The gender distribution of the patients was 662 men and 204 women. The age of enrolled patients ranged from 56 to 63 years old, and the demographic data were similar in the two groups. The majority of patients enrolled in these studies had standard clinical, endoscopic, and histological diagnoses of esophageal carcinoma (esophagus or gastroesophageal junction). The pathological types included 408 squamous cell carcinomas and 458 adenocarcinomas, with two studies that compared neoadjuvant chemotherapy with neoadjuvant chemoradiotherapy in patients with a pathological subtype of either esophageal adenocarcinoma or squamous cell carcinoma.

**Table 1:** Characteristics of clinical trials of neoadjuvant chemotherapy versus neoadjuvant chemoradiotherapy.

Author(ref)	Year	Group	Number of patients	Median (range) age(years)	Pathological subtype	Stage	Follow-up	Neoadjuvant treatment schedule
M.Stahl et al.	2017	Neoadjuvant chemotherapy	59	56.0	Esophageal adenocarcinoma	T3-4, I-III	> 3 years	2.5 courses of cisplatin (50mg/m2), fluorouracil (2g/m2), and leucovorin (500mg/m2)
		Neoadjuvant chemoradiotherapy	60	60.6				Two courses of cisplatin (50mg/m2), fluorouracil (2g/m2), and leucovorin (500mg/m2), and concurrent chemotherapy cisplatin (50mg/m2), day 1+8 and etoposide (80mg/m2) days 3–5 to a total dose of 30 Gy given at 2.0 Gy/fraction, 5 fractions/week
F.Klevebro et al.	2016	Neoadjuvant chemotherapy	66	63.0	Esophageal adenocarcinoma	T1-3, any N	> 3 years	Three cycles of cisplatin, 100mg/m2 on day 1 and fluorouracil 750 mg/m2/24h on day 1–5. Each cycle lasted 21 days
		Neoadjuvant chemoradiotherapy	65					40 Gy was given (2 Gy once daily in 20 fractions, 5 days a week) concomitant with chemotherapy cycles 2 and 3
		Neoadjuvant chemotherapy	25	63.0	Oesophageal squamous cell carcinoma			Three cycles of cisplatin 100mg/m2 on day 1 and fluorouracil 750 mg/m2/24h, on day 1–5. Each cycle lasted 21 days
		Neoadjuvant chemoradiotherapy	25					40 Gy was given (2 Gy once daily in 20 fractions, 5 days a week) concomitant with chemotherapy cycles 2 and 3
Burmeister et al.	2011	Neoadjuvant chemotherapy	36	63(36–75)	Esophageal adenocarcinoma	T2-3N0-1	Median: 94 months	Cisplatin (80mg/m2) and influsional 5-fluorouracil (1000mg/m2/day) on day 1 and 21
		Neoadjuvant chemoradiotherapy	39	60(41–73)				The sane drugs accompanied by concurrent radiation therapy commencing on day 21 of chemotherapy and 5-fluorouracil reduced to 800 mg/m2/day, and 35 Gy in 15 fractions over 3 weeks
Swisher SG et al.	2010	Neoadjuvant chemotherapy	76	59(23–77)	Esophageal adenocarcinoma (n = 133) Oesophageal squamous cell carcinoma (n = 24)	T1-3N0-1	> 3 years	3 courses of cisplatin, fluorouracil or 3–5 courses of cisplatin, fluorouracil + arabinoside
		Neoadjuvant chemoradiotherapy	81	58(38–74)				2 courses of chemotheraoy consisting of 5-fluorouracil, cisplatinum, paclitaxel + 45 Gy radiation therapy in 25 fractions + 5-fluorouracil, cisplatinu, or 2 courses of cisplatin + CPT-11 +45 Gy radiation therapy in 25 fractions + 5-fluorouracil, cisplatinum
Cao et al.	2009	Neoadjuvant chemotherapy	119	Not reported	Oesophageal squamous cell carcinoma	Ⅱ/Ⅲ/Ⅳ	> 3 years	Cisplatin (20mg/m2/day) +5-fliorouracil (500mg/m2/day) +mitomycin (10mg/m2/day) regimen
		Neoadjuvant chemoradiotherapy	118					Cisplatin (20mg/m2/day) +5-fliorouracil (500mg/m2/day) +mitomycin (10mg/m2/day) regimen, and daily fractions of 2 Gy (days 1–5,8–12,15–19, and 22–26) to a total dose of 40 Gy
Nygaard et al	1992	Neoadjuvant chemotherapy	50	62.9(44–77)	Oesophageal squamous cell carcinoma	T1-2NxM0	> 3 years	Two cycles of cisplatin (100mg/m2/cycle) and bleomycin (50mg/m2/cycle)
		Neoadjuvant chemoradiotherapy	47	60.1(50–74)				Two cycles of cisplatin (100mg/m2/cycle) and bleomycin (50mg/m2/cycle), and 35 Gy in 20 fractions

The tumor stage of most patients ranged from I to III according to the American Joint Committee on Cancer tumor-node-metastasis (TNM) staging system [27]. Of the six trials included in the meta-analysis, patients in most of the studies were clinical stage II/III patients, while one study enrolled patients in clinical stage IV [25].

### Methodological assessment of study quality

**Fig 2:**
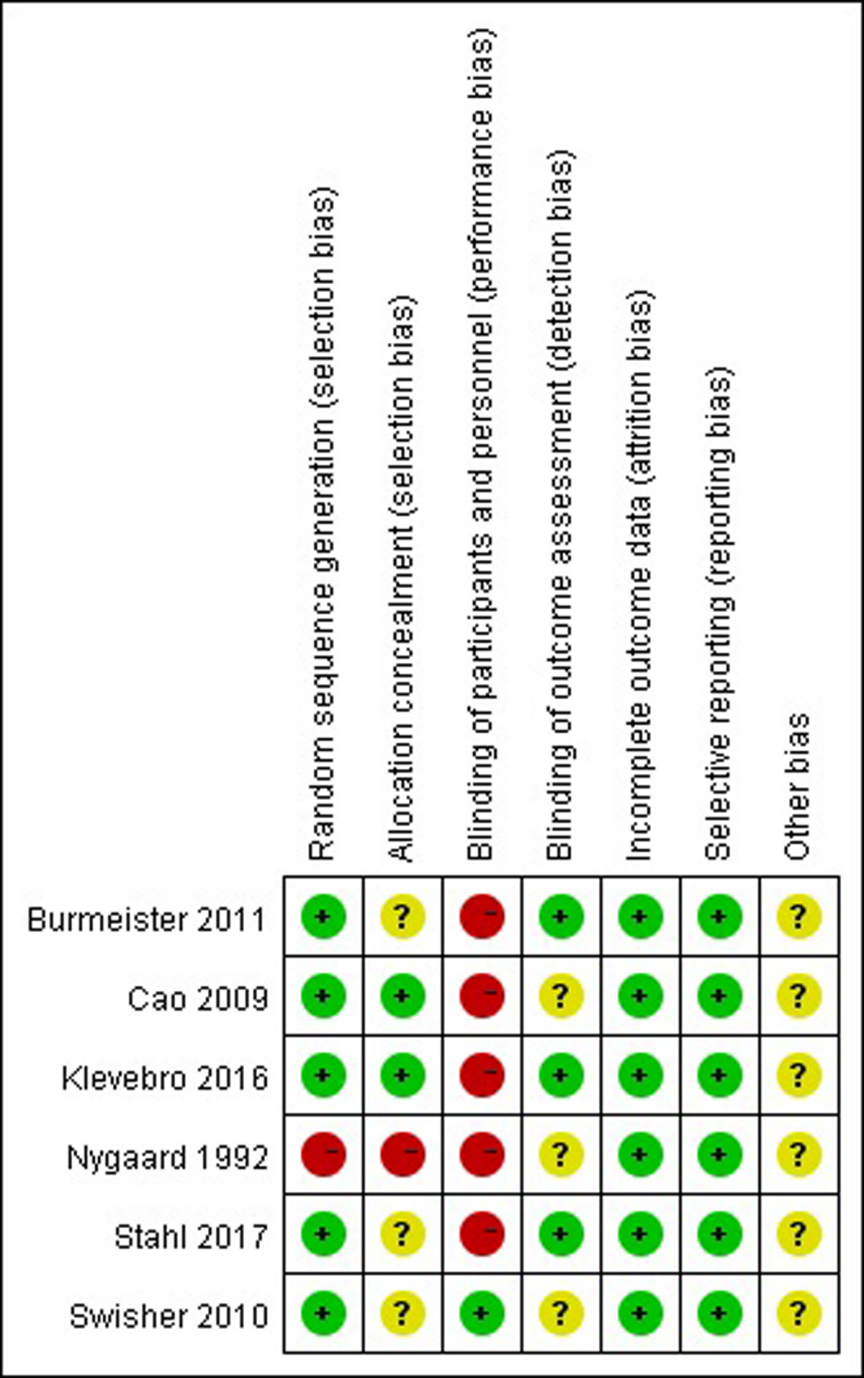
Risk of bias summary: This risk of bias tool incorporates the assessment of randomization (sequence generation and allocation concealment), blinding (participants and outcome assessors), incomplete outcome data, and selective outcome reporting and other risk of bias. The items were judged as “low risk” “unclear risk” or “high risk”, where red means “high risk”, green means “low risk” and yellow means “unclear risk”.

**Fig 3:**
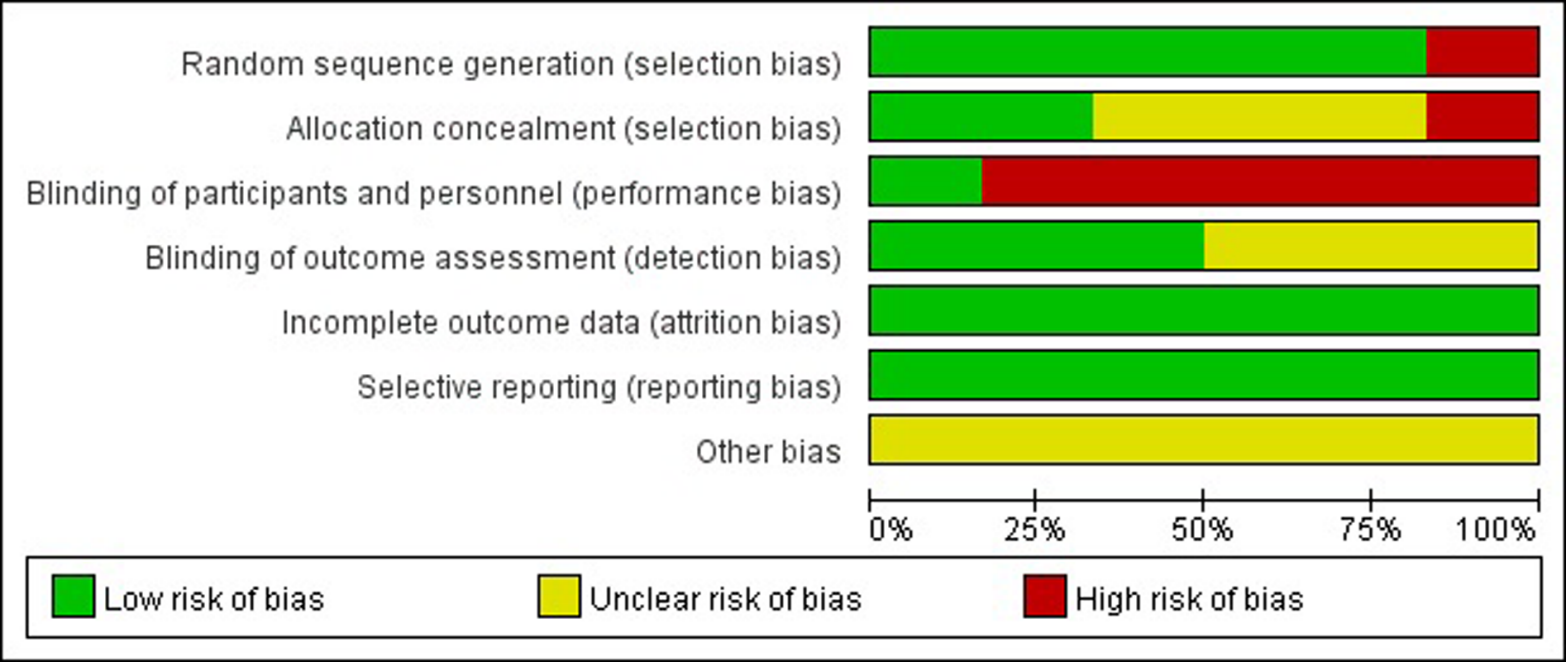
Risk of bias graph exhibiting the review of the authors’ judgments about each risk of bias item, presented as percentages across all included studies.

The methodological quality assessment of the six included studies is presented in Fig 2. The quality of these studies was low to moderate. The eligible studies included six RCTs [21–26]. Only four studies adopted random sequence generation [21–24]. Three studies [21–23] reported that enrolled patients were randomized through the use of a computerized randomization program. None of the studies referenced the details of allocation concealment, which gave rise to high risks of selection and measurement bias. None of the included studies mentioned blinded status, as it is impossible to blind patients who receive the treatment. All trials reported important outcomes and thus had a low risk of reporting bias and incomplete data. The sample size, follow-up time, sub-type of pathological and clinical stage of carcinoma, and different centers with different neoadjuvant treatment schedules, which contributed to high risks of selection and measurement bias, may have affected the results. (Fig 3)

### Primary outcomes

#### 3- and 5-year survival rate

**Fig 4:**
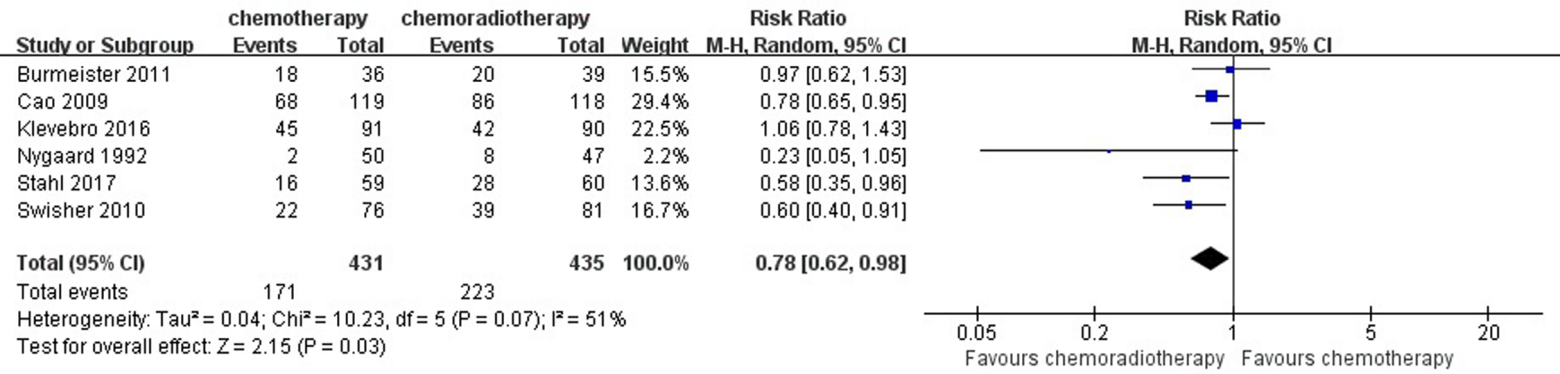
Forest plot of the included studies for 3-year survival. M-H, Mantel-Haenszel.

**Fig 5:**
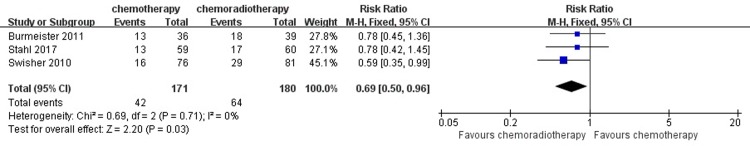
Forest plot of the included studies for 5-year survival. M-H, Mantel-Haenszel.

Data regarding 3-year survival was available in six trials with 866 patients. One hundred seventy-one out of 431 patients (39.68%) achieved 3-year survival in the neoadjuvant chemotherapy group, while 223 out of 435 patients (51.26%) in neoadjuvant chemoradiotherapy group achieved 3-year survival. Significant differences were found in 3-year survival between the two groups (I^2^ = 51%, RR = 0.78, 95% CI = 0.62–0.98, P = 0.03). Three studies reported the 5-year survival. In the neoadjuvant chemotherapy group, 42 out of 171 patients (24.56%) achieved 5-year survival and 64 out of 180 patients (35.56%) in the neoadjuvant chemoradiotherapy group achieved 5-year survival. The 5-year survival analysis indicated that there was an obvious difference between the two groups (I^2^ = 0%, RR = 0.69, 95% CI = 0.50–0.96, P = 0.03). It indicates that neoadjuvant chemoradiotherapy can significantly increase long-term survival, including 3-year and 5-year survival compared with the neoadjuvant chemotherapy group. Therefore, the 3-year and 5-year survival rate were significantly higher in patients who were treated with neoadjuvant chemoradiotherapy than those in patients treated with neoadjuvant chemotherapy. (Figs 4 and 5)

### Secondary outcomes

#### R0 resection

**Fig 6:**
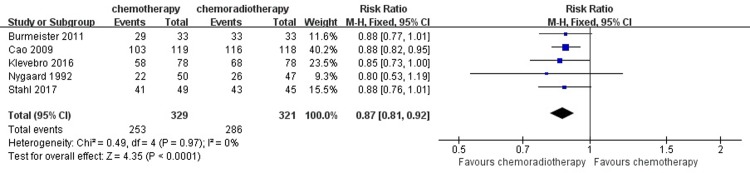
Forest plot of the included studies for R0 resection. M-H, Mantel-Haenszel.

From the included studies, to assess patients’ rate of R0 resection remission, we enrolled a total of 650 patients who underwent surgical resection: 329 patients in the neoadjuvant chemotherapy group and 321 in the neoadjuvant chemoradiotherapy group. Based on a summary of the data from each study, 386 (89.10%) patients in the neoadjuvant chemoradiotherapy group achieved R0 resection, compared with 253 (76.90%) patients in the neoadjuvant chemotherapy group. A heterogeneity test revealed low heterogeneity among the studies (I^2^ = 0%); thus, the fixed-effects model was used. Compared with the neoadjuvant chemotherapy group, the pooled RR for the concurrent neoadjuvant chemoradiotherapy group was 0.87 (95% CI = 0.81–0.92, P < 0.0001). The analysis of the R0 resection indicated that the patients in the neoadjuvant chemoradiotherapy group had a significantly higher rate of R0 resection. (Fig 6)

#### Pathological complete response

**Fig 7:**
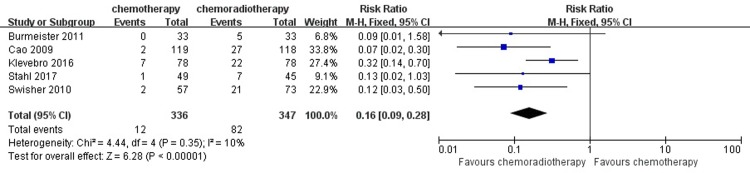
Forest plot of the included studies for pathological complete response. M-H, Mantel-Haenszel.

Five studies with 683 patients reported the result of pathological complete response. Twelve out of 336 patients (3.57%) achieved pathological complete response in the neoadjuvant chemotherapy group, while 82 out of 347 patients (23.63%) in the neoadjuvant chemoradiotherapy group achieved pathological complete response. For this result, the low heterogeneity among the studies was revealed (I^2^ = 10%), so the fixed-effects model was adopted. A pooled analysis revealed that there was a significant difference between the neoadjuvant chemotherapy and neoadjuvant chemoradiotherapy groups in inducing pathological complete response (RR = 0.16, 95% CI = 0.09–0.28, P < 0.00001). (Fig 7)

#### Perioperative mortality and postoperative complication

**Fig 8:**
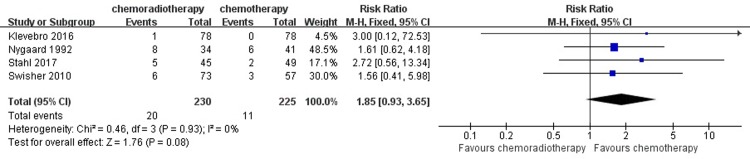
Forest plot of the included studies for perioperative mortality. M-H, Mantel-Haenszel.

Four studies, which included a total of 455 patients with 225 patients in the neoadjuvant chemotherapy group and 230 in the neoadjuvant chemoradiotherapy group, reported a rate of perioperative mortality. Eleven out of 225 patients (4.89%) suffered perioperative mortality in the neoadjuvant chemotherapy group, while 20 out of 230 patients (8.70%) suffered perioperative mortality in the neoadjuvant chemoradiotherapy group. A heterogeneity test revealed a low heterogeneity among the studies (I^2^ = 0%), so the fixed-effects model was adopted. There was no significant difference between the neoadjuvant chemotherapy and the neoadjuvant chemoradiotherapy in inducing perioperative mortality (RR = 1.85, 95% CI = 0.93–3.65, P = 0.08). (Fig 8)

#### Postoperative complications

For the outcome of postoperative complications, we mainly reported the incidence of pulmonary complications, cardiac complications and anastomotic leak in detail with the limited data.

#### Pulmonary complications

**Fig 9:**
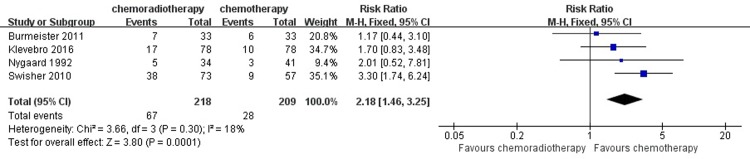
Forest plot of the included studies for pulmonary complications. M-H, Mantel-Haenszel.

In our meta-analysis, pulmonary complications included pneumonia, pleural effusion requiring postoperative placement of drains, and respiratory failure in general. Four studies summarized the incidence of pulmonary complications. The analysis using the fixed-effect model pooled estimate of RR was 2.18 (95% CI = 1.46–3.25, P = 0.0001), which showed a significant difference between the two groups. The relevant details are shown in Fig 9.

#### Cardiovascular complications

**Fig 10:**
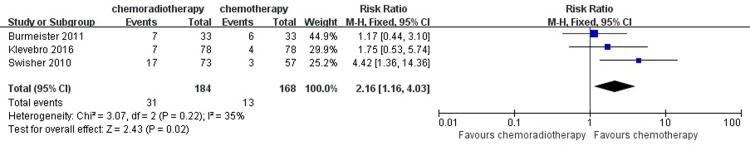
Forest plot of the included studies for cardiovascular complications. M-H, Mantel-Haenszel.

Cardiovascular complications included cardiac arrhythmias requiring treatment, myocardial infarction, cerebral embolism, and pulmonary embolism. A pooled analysis revealed that there was a statistically significant difference between the two groups in the incidence of cardiovascular complications (RR = 2.16, 95% CI = 1.16–4.03, P = 0.02). (Fig 10)

#### Anastomotic leak

**Fig 11:**
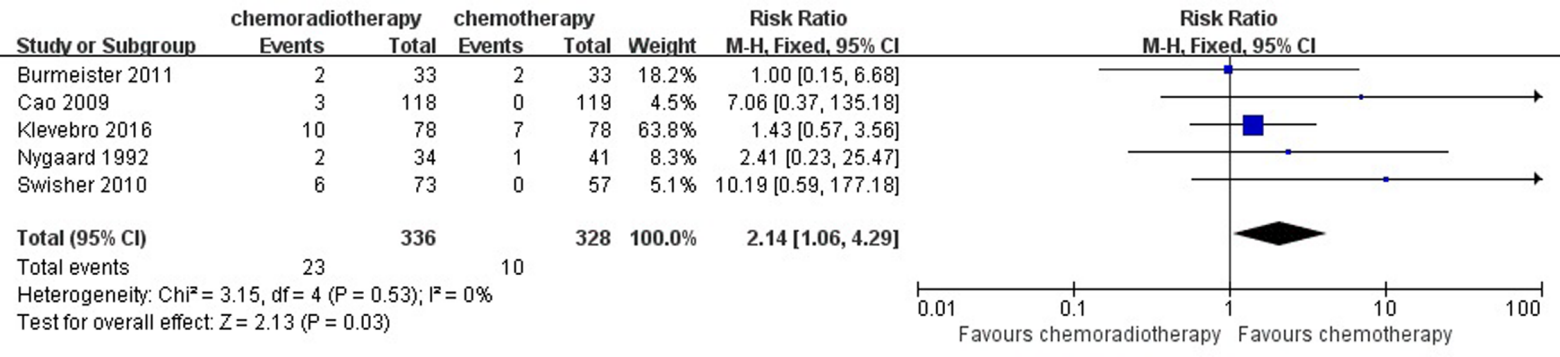
Forest plot of the included studies for anastomotic leak. M-H, Mantel-Haenszel.

Anastomotic leakage was assessed using a computed tomography (CT) scan with an oral water-soluble contrast medium, and any uncertainty was followed up with endoscopy. The pooled analysis under the fixed-effects model indicated that there was a significant difference in the incidence of anastomotic leakage between the neoadjuvant chemotherapy and neoadjuvant chemoradiotherapy groups (RR = 2.14, 95% CI = 1.06–4.29, P = 0.03). (Fig 11)

#### Hospital stay

**Fig 12:**
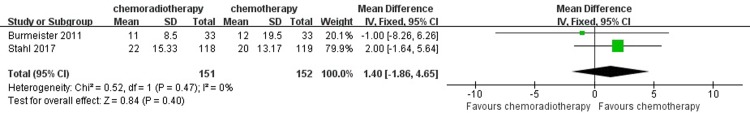
Forest plot of the included studies for hospital stay. M-H, Mantel-Haenszel.

For the result of the hospital stay between the two groups, the analysis using the fixed-effect model pooled estimate of RR was 1.40 (95% CI = -1.86–4.65, P = 0.40), which showed no significant difference between the neoadjuvant chemotherapy and neoadjuvant chemoradiotherapy treatment strategy. (Fig 12)

### Subgroup analysis

**Fig 13:**
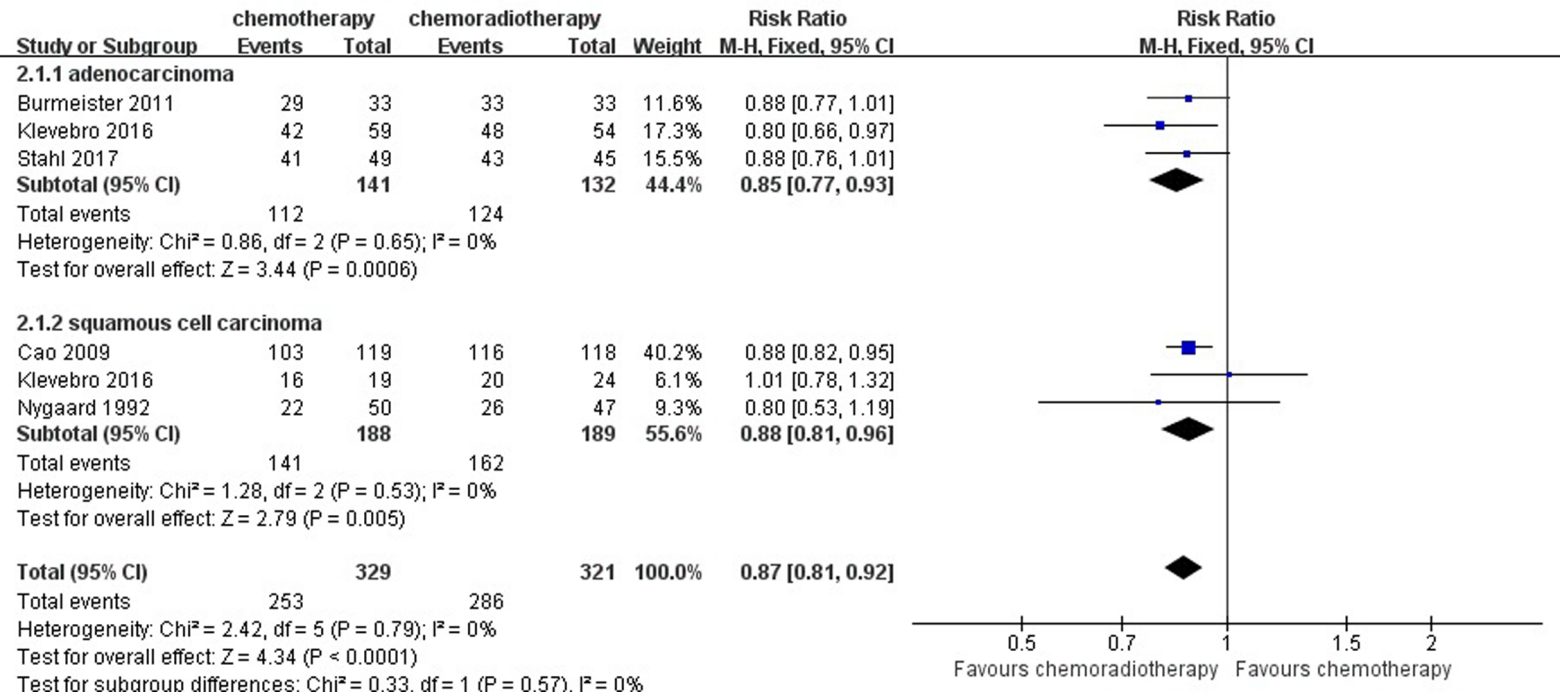
Forest plot of the included studies for R0 resection in two histopathologies of the tumor (adenocarcinoma or squamous cell carcinoma). M-H, Mantel-Haenszel.

**Fig 14:**
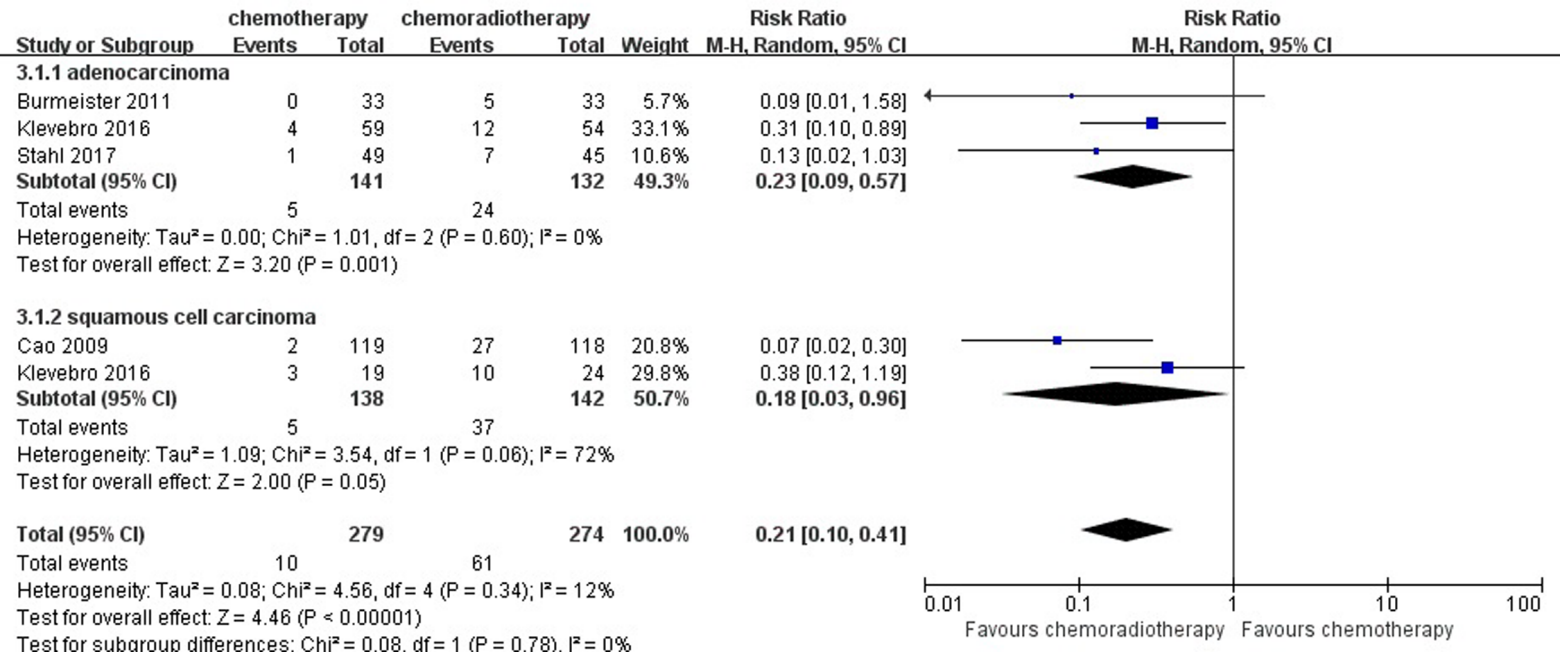
Forest plot of the included studies for pathological complete response in two histopathologies of the tumor (adenocarcinoma or squamous cell carcinoma). M-H, Mantel-Haenszel.

Furthermore, we then performed a subgroup analysis to compare neoadjuvant chemotherapy and neoadjuvant chemoradiotherapy in the same histopathology of the tumor (adenocarcinoma or squamous cell carcinoma) in the available results of R0 resection and pathological response. Significant results were observed both in the adenocarcinoma and squamous cell carcinoma subgroups (RR = 0.85, 95% CI = 0.77–0.93, P = 0.0006; RR = 0.88, 95% CI = 0.81–0.96, P = 0.005). For the result of pathological complete response in the adenocarcinoma and squamous cell carcinoma subgroups, there was an obvious difference between the neoadjuvant chemotherapy and neoadjuvant chemoradiotherapy group (RR = 0.23, 95% CI = 0.09–0.57, P = 0.001; RR = 0.18, 95% CI = 0.03–0.96, P = 0.05). (Figs 13 and 14)

### Sensitivity analysis and publication bias

**Fig 15:**
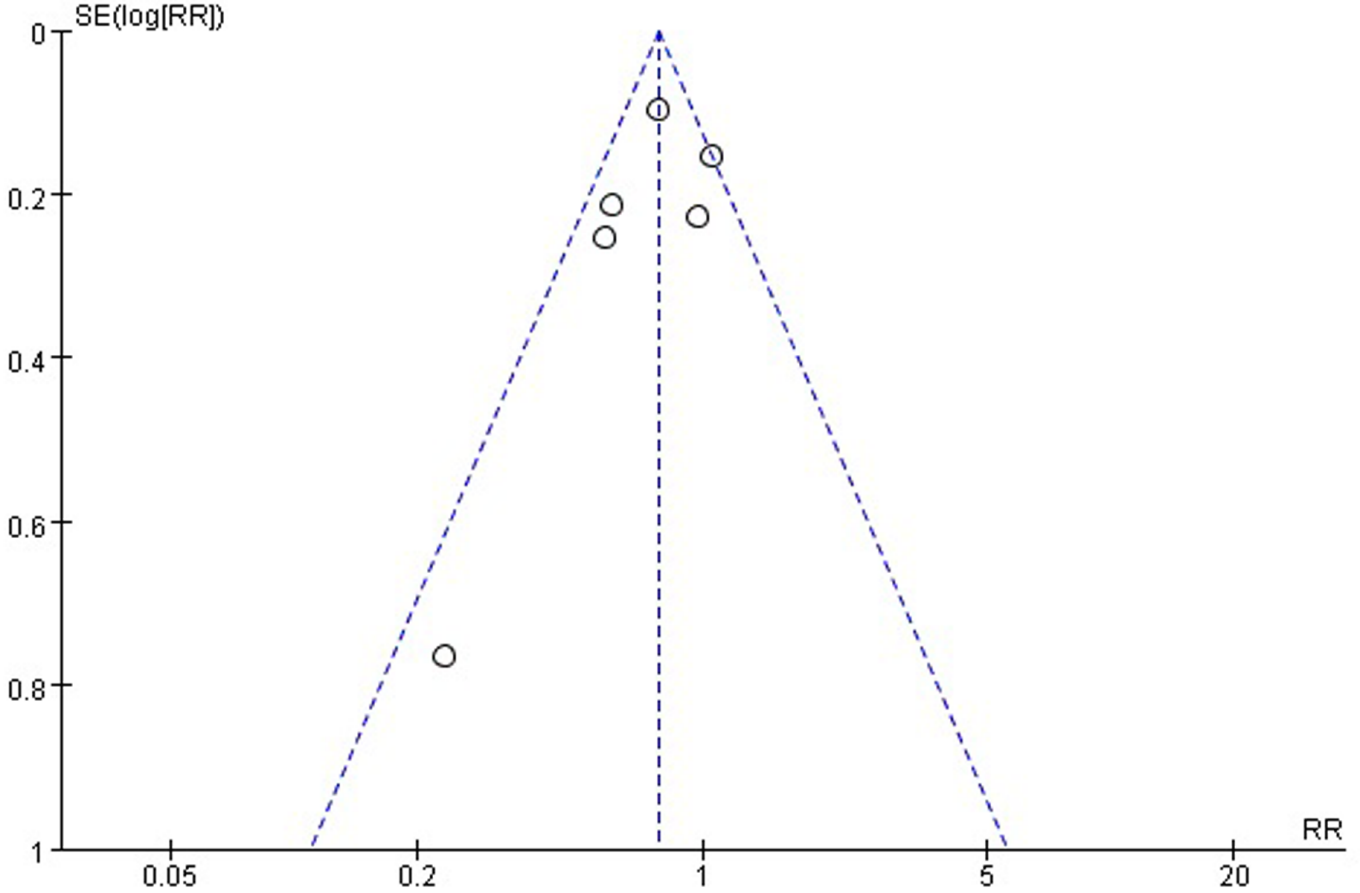
Funnel plots of the included studies for 3-year survival. RR, risk ratio; SE, standard error.

The quality of the studies included in the meta-analysis was low to moderate, thus a sensitivity analysis was performed to assess the stability of pooled results. The sequential removal of each study did not change the outcomes of the primary overall analysis. A funnel plot of clinical trials reporting 3-year survival outcomes is shown in Fig 15. Publication bias may exist but was not apparent; thus, the affected quantity in the combined effect is small. The result is discussed later.

## Discussion

### Summary of main results

Surgery has always been considered the standard treatment for patients with resectable esophageal cancer, but the local recurrence rate after the operation is as high as 40%-60%, and the overall survival rate of 5 years is merely 30% [28,29]. Based on the accumulating evidence indicating that the new adjuvant therapy has systemic and local therapy effects and that the two may have a synergistic effect, it has been more recognized in the treatment of locally advanced esophageal cancer. As few sample studies have addressed these treatments, the roles of neoadjuvant chemoradiotherapy and neoadjuvant chemotherapy in treating esophageal cancer remain controversial. The purpose of neoadjuvant therapy is to reduce the tumor size and maximize local control by using the radiation sensitization of chemotherapy. Although neoadjuvant therapy may have a satisfactory clinical efficacy, the role of neoadjuvant chemotherapy and neoadjuvant chemoradiotherapy in clinical practice is not yet well established.

In this study, we identified six RCTs that investigated the primary and secondary outcomes associated with neoadjuvant chemoradiotherapy and neoadjuvant chemotherapy interventions. Our meta-analysis showed that neoadjuvant chemoradiotherapy should be recommended with a long-term survival benefit in patients with esophagus or gastroesophageal junction cancer, as well as a significant high rate of R0 resection and pathological complete response. A subgroup analysis performed between different histopathologies of the tumor yielded the same results.

The primary outcome of 3-year and 5-year survival in the six included studies should be discussed. Our study reported that the 3-year and 5-year survival rates were significantly higher in patients who were treated with neoadjuvant chemoradiotherapy than in those treated with neoadjuvant chemotherapy. However, the heterogeneity between studies was high for the 3-year survival analysis and then decreased again to 0% for the 5-year survival analysis. There were six studies in total that reported the 3-year survival. This heterogeneity may primarily be associated with the clinical heterogeneity of different studies. First, from the articles we included, the earliest study was conducted from 1983 to 1988 and enrolled patients with tumor stage T1 or T2, NX and M0 (26). However, with the development of medical research, Barratt surveillance was introduced during these years in the West; thus, the rate of 3-year survival was higher in other included studies. In addition, the results of the analysis of the three-year survival rate were also influenced by the age distribution of the population in different studies and the severity of the disease.

For the secondary outcome of R0 resection, some studies have shown that neoadjuvant chemotherapy and radiotherapy can reduce esophageal cancer staging, improve the R0 resection rate and achieve complete remission of pathology, thus improving prognosis [3,30,31]. Previous research results show that the complete remission rate after R0 resection and neoadjuvant therapy is an independent prognostic factor to improve the long-term survival rate and decrease the local recurrence rate of esophageal carcinoma [32,33]. Therefore, it is significant to explore the effect of the two treatment methods on the rate of R0 resection. In this meta-analysis, neoadjuvant chemoradiotherapy had a significant advantage in a higher rate of R0 resection and pathological complete response compared with those in patients in the neoadjuvant chemotherapy group.

The results of perioperative mortality should also be discussed. Perioperative mortality, defined as death within 1 month after operation, occurred in 20 patients in the neoadjuvant chemotherapy group and in 11 patients in the chemoradiotherapy group. The rate of perioperative mortality was based on patients undergoing esophageal resection. Only two studies showed the detailed information of perioperative mortality. F. Klevebro’s study demonstrated that perioperative mortality was increased in the chemoradiotherapy group (2 of 52 patients in chemotherapy group [3.8%] and 5 of 49 in chemoradiotherapy group [10.2%]); causes for death were pneumonia, anastomotic leakage and cardiac disease [22]. Similarly, in Knut Nygaard’ s clinical trial, pulmonary complications were the dominant cause of perioperative deaths; in the view of authors the respiratory failure leading to death was likely related to bleomycin treatment [26]. In our meta-analysis, there were no significant differences between the two groups in perioperative mortality (RR = 1.85, 95% CI = 0.93–3.65, P = 0.08). For the result of perioperative mortality, finding that the lower confidence limit for the RR barely exceeded 1.0 and that the horizontal block lay to the right of the vertical line indicated that the treatment of chemotherapy may have achieved a lower rate of perioperative mortality.

The postoperative complications noted in the five included studies should be discussed. In our study, postoperative complications were based on patients undergoing esophageal resection. One study categorized the complications as surgical complications and nonsurgical complications, and provided a detailed definition [21]. Anastomotic leakage, conduit necrosis, bleeding, chylothorax and recurrent laryngeal nerve paralysis were defined as surgical complications. Other complications, such as cardiovascular complications, respiratory failure and infections, which were not related to the operation, were identified as nonsurgical complications. Three studies reported postoperative complications, including surgical and nonsurgical complications [21,23,25]. Two studies reported surgical adverse events, including pulmonary, cardiovascular and gastrointestinal events in total [24,26]. For the result of postoperative complications, we mainly reported the incidence of pulmonary complications, cardiac complications and anastomotic leak in detail with the limited data. However, care should be taken with neoadjuvant chemoradiotherapy in patients, for whom there is an increased risk of postoperative complications, especially pulmonary complications were apparent. For anastomotic leak and cardiac complications, neoadjuvant chemotherapy had a comparable effect to neoadjuvant chemoradiotherapy for patients who underwent an operation. A study demonstrated that with a daily dose of up to 40 Gy of radiation therapy, the toxicity of radiation would increase, which might be a critical factor in low postoperative complications advantages [34]. Reportedly, neoadjuvant chemoradiotherapy may increase the incidence of postoperative complications [35]. Furthermore, as surgical techniques have improved, perioperative mortality and complications decrease [36]. Therefore, it is critically important to determine how to maintain the R0 resection and long-term benefits and reduce the perioperative mortality and complication rates of patients with a neoadjuvant chemoradiotherapy strategy. High-quality randomized trials with large sample sizes are needed for confirmation.

### Comparison with previous studies

The number of clinical studies directly comparing neoadjuvant chemotherapy with neoadjuvant chemoradiotherapy is very rare and limited. In 2017, a recently completed systematic review and network meta-analysis of neoadjuvant therapy combined with surgery for patients with resectable esophageal squamous cell carcinoma (ESCC) investigated the effect of the two groups indirectly, showing that neoadjuvant chemoradiotherapy might be the best choice for resectable ESCC because it could increase the radical resection rate and lower the occurrence of complications, thereby prolonging survival time [33]. A study by Deng HY et al., including five RCTs with 709 patients with esophageal cancer who were enrolled until March 31, 2016, demonstrating the use of neoadjuvant therapy for treating esophageal cancer, suggested that esophageal squamous cell carcinoma responds better to neoadjuvant chemoradiotherapy, whereas esophageal adenocarcinoma responds best to neoadjuvant chemotherapy alone to avoid the adverse effects of radiation [15]. Another prospective study in 2017 that investigated the role of neoadjuvant chemotherapy and radiation treatment in resectable esophageal cancer advocated for neoadjuvant chemotherapy alone followed by radical esophageal resection [37]. The influential factors of the results of the neoadjuvant chemotherapy and chemoradiotherapy were intricate, such as the systemic condition of patients, the manner of administration, and the operations [38]. In our study, neoadjuvant chemoradiotherapy could benefit patients with esophageal squamous cell carcinoma and esophageal adenocarcinoma. A study by Mengying Fan et al. supported the view that, compared with neoadjuvant chemotherapy, induction neoadjuvant chemoradiotherapy could achieve a long-term survival benefit in patients with esophageal carcinoma [16]. With a similar conclusion, our study analyzed more detailed and meaningful indicators, such as the patient's 3- and 5-year survival, complications, and subgroup analysis of the patients to support our conclusions.

To our knowledge, this is an updated meta-analysis to compare the effects of neoadjuvant chemotherapy and neoadjuvant chemoradiotherapy interventions followed by surgery for cancer of the esophagus or gastroesophageal junction. However, many of the clinical trials enrolled small numbers of patients, and it is difficult to detect a treatment benefit through these meta-analyses, even if a benefit actually exists. After comprehensively searching the databases, we found six qualified RCTs with a total of 866 patients. With this larger sample size, we were able to perform both an overall analysis for cancer of the esophagus or gastroesophageal junction, perioperative mortality, and postoperative complications and separate subgroup analyses for esophageal adenocarcinoma and squamous cell carcinoma. Previous meta-analyses did not classify and provide detailed descriptions of the complications of the therapies. To date, this meta-analysis included the latest clinical trials, which are currently the direct comparison of neoadjuvant chemotherapy and neoadjuvant chemoradiotherapy with a relatively larger sample size and a wider distribution of patients and pathologic types. Moreover, in our analysis of the results, we clarified the postoperative complications into respiratory complications, cardiovascular complications and anastomotic leakage, which other studies lack. To evaluate possible sources of heterogeneity, we also performed a subgroup analysis of different histological types and performed a sensitivity analysis to assess the influence of each study on the overall pooled estimate.

### Limitations of the study

Certain limitations of our meta-analysis should be noted. First, the foremost limitation is the scarcity of high-quality, multicenter, large-sample standard RCTs with which to directly compare the two neoadjuvant strategies. Thus, more such trials are needed to verify the outcomes of this meta-analysis. Second, a significant statistical heterogeneity of the primary outcome of postoperative complications and pathological complete response in the squamous cell carcinoma subgroup among the included trials was observed, which may be explained by the clinical diversity among trials, the small subgroup sample sizes, differences in chemotherapy, chemoradiotherapy dose or surgical procedures in the included studies. Additionally, it is understandable that from an Eastern hemisphere perspective squasmous cell carcinoma is far more abundant than adenocarcinoma; it is the opposite in the Western hemisphere, and most of the clinical trials have been performed in the Western hemisphere. In the west, Barratt surveillance results in an abundance of high grade dysplagia, clinical stage 1, and inner stage 2 disease that uncommonly metastasizes to regional lymph noded, and thus has a good prognosis with ablative or surgical therapy without neoadjuvant therapy. These conditions may introduce a publication bias. Moreover, there is no uniformity of the description of the complications, and these trials seldom provided details of the randomized techniques and allocation concealment. These issues may produce selection bias and measurement bias, which may affect the results.

## Conclusions

In conclusion, neoadjuvant chemoradiotherapy was recommended with a long-term survival benefit in patients with esophagus or gastroesophageal junction cancer. Patients who underwent the treatment of neoadjuvant chemoradiotherapy could achieve a high rate of R0 resection and pathological complete response as well. However, care should be taken because of an increased risk of postoperative complications, especially pulmonary and cardiac complications and anastomotic leak, which were apparent. Future trials should include modern staging methods to facilitate the appropriate stratification of patients and measures for assessing the quality of surgery. In view of the heterogeneity and different follow-up times, whether these conclusions are applicable should be further determined in future studies.

## Supporting information

S1 File PRISMA checklist. (DOC)

S2 File Excluded full-text articles. (DOC)
