# Supplementary material for: Neoadjuvant chemotherapy versus neoadjuvant chemoradiotherapy for cancer of the esophagus or the gastroesophageal junction: A meta-analysis based on clinical trials
Source: PLoS One. 2018 Aug 23;13(8):e0202185. doi: 10.1371/journal.pone.0202185 (PMC6107145; doi:10.1371/journal.pone.0202185)
Supplement: S2 File — (DOC) [file pone.0202185.s002.doc]

**37 potentially eligible articles retrieved with full text for more detailed screening and 31 full-text articles were excluded**

**20 studies with comparison not related to the topic:**

1. Mukherjee S, Hurt CN, Gwynne S, Sebag-Montefiore D, Radhakrishna G, Gollins S, Hawkins M, Grabsch HI, Jones G, Falk S, Sharma R, Bateman A, Roy R, Ray R, Canham J, Griffiths G, Maughan T, Crosby T. NEOSCOPE: A randomised phase II study of induction chemotherapy followed by oxaliplatin/capecitabine or carboplatin/paclitaxel based pre-operative chemoradiation for resectable oesophageal adenocarcinoma. Eur J Cancer. 2017 Mar;74:38-46. doi: 10.1016/j.ejca.2016.11.031. Epub 2017 Feb 8. PubMed PMID: 28335886;

2. Hoeppner J, Lordick F, Brunner T, Glatz T, Bronsert P, Röthling N, Schmoor C, Lorenz D, Ell C, Hopt UT, Siewert JR. ESOPEC: prospective randomized controlled multicenter phase III trial comparing perioperative chemotherapy (FLOT protocol) to neoadjuvant chemoradiation (CROSS protocol) in patients with adenocarcinoma of the esophagus (NCT02509286). BMC Cancer. 2016 Jul 19;16:503. doi: 10.1186/s12885-016-2564-y. PubMed PMID: 27435280.

3. van der Woude SO, Hulshof MC, van Laarhoven HW. CROSS and beyond: a clinical perspective on the results of the randomized ChemoRadiotherapy for Oesophageal cancer followed by Surgery Study. Chin Clin Oncol. 2016 Feb;5(1):13. doi: 10.3978/j.issn.2304-3865.2016.02.04. PubMed PMID: 26932437.

4. Robb WB, Messager M, Dahan L, Mornex F, Maillard E, D'Journo XB, Triboulet JP, Bedenne L, Seitz JF, Mariette C; Fédération Francophone de Cancérologie Digestive; Société Française de Radiothérapie Oncologique; Union des Centres de Lutte Contre le Cancer; Groupe Coopérateur Multidisciplinaire en Oncologie; French EsoGAstric Tumour working group - Fédération de Recherche En Chirurgie. Patterns of recurrence in early-stage oesophageal cancer after chemoradiotherapy and surgery compared with surgery alone. Br J Surg. 2016 Jan;103(1):117-25. doi: 10.1002/bjs.9959. Epub 2015 Oct 29. PubMed PMID: 26511668.

5. Shapiro J, van Lanschot JJB, Hulshof MCCM, van Hagen P, van Berge Henegouwen MI, Wijnhoven BPL, van Laarhoven HWM, Nieuwenhuijzen GAP, Hospers GAP, Bonenkamp JJ, Cuesta MA, Blaisse RJB, Busch ORC, Ten Kate FJW, Creemers GM, Punt CJA, Plukker JTM, Verheul HMW, Bilgen EJS, van Dekken H, van der Sangen MJC, Rozema T, Biermann K, Beukema JC, Piet AHM, van Rij CM, Reinders JG, Tilanus HW, Steyerberg EW, van der Gaast A; CROSS study group. Neoadjuvant chemoradiotherapy plus surgery versus surgery alone for oesophageal or junctional cancer (CROSS): long-term results of a randomised controlled trial. Lancet Oncol. 2015 Sep;16(9):1090-1098. doi: 10.1016/S1470-2045(15)00040-6. Epub 2015 Aug 5. PubMed PMID: 26254683.

6. Vincent J, Mariette C, Pezet D, Huet E, Bonnetain F, Bouché O, Conroy T, Roullet B, Seitz JF, Herr JP, Di Fiore F, Jouve JL, Bedenne L; Fédération Francophone de Cancérologie Digestive (FFCD). Early surgery for failure after chemoradiation in operable thoracic oesophageal cancer. Analysis of the non-randomised patients in FFCD 9102 phase III trial: Chemoradiation followed by surgery versus chemoradiation alone. Eur J Cancer. 2015 Sep;51(13):1683-93. doi: 10.1016/j.ejca.2015.05.027. Epub 2015 Jul 7. PubMed PMID: 26163097.

7. Fernandez-Martos C, Garcia-Albeniz X, Pericay C, Maurel J, Aparicio J, Montagut C, Safont MJ, Salud A, Vera R, Massuti B, Escudero P, Alonso V, Bosch C, Martin M, Minsky BD. Chemoradiation, surgery and adjuvant chemotherapy versus induction chemotherapy followed by chemoradiation and surgery: long-term results of the Spanish GCR-3 phase II randomized trial†. Ann Oncol. 2015 Aug;26(8):1722-8. doi: 10.1093/annonc/mdv223. Epub 2015 May 8. PubMed PMID: 25957330.

8. Hong YS, Nam BH, Kim KP, Kim JE, Park SJ, Park YS, Park JO, Kim SY, Kim TY, Kim JH, Ahn JB, Lim SB, Yu CS, Kim JC, Yun SH, Kim JH, Park JH, Park HC, Jung KH, Kim TW. Oxaliplatin, fluorouracil, and leucovorin versus fluorouracil and leucovorin as adjuvant chemotherapy for locally advanced rectal cancer after preoperative chemoradiotherapy (ADORE): an open-label, multicentre, phase 2, randomised controlled trial. Lancet Oncol. 2014 Oct;15(11):1245-53. doi: 10.1016/S1470-2045(14)70377-8. Epub 2014 Sep 4. PubMed PMID: 25201358.

9. Mariette C, Dahan L, Mornex F, Maillard E, Thomas PA, Meunier B, Boige V, Pezet D, Robb WB, Le Brun-Ly V, Bosset JF, Mabrut JY, Triboulet JP, Bedenne L, Seitz JF. Surgery alone versus chemoradiotherapy followed by surgery for stage I and II esophageal cancer: final analysis of randomized controlled phase III trial FFCD 9901. J Clin Oncol. 2014 Aug 10;32(23):2416-22. doi: 10.1200/JCO.2013.53.6532. Epub 2014 Jun 30. PubMed PMID: 24982463.

10. Shridhar R, Freilich J, Hoffe SE, Almhanna K, Fulp WJ, Yue B, Karl RC, Meredith K. Single-institution retrospective comparison of preoperative versus definitive chemoradiotherapy for adenocarcinoma of the esophagus. Ann Surg Oncol. 2014 Nov;21(12):3744-50. doi: 10.1245/s10434-014-3795-2. Epub 2014 May 23. PubMed PMID: 24854492.

11. Jeong SY, Park JW, Nam BH, Kim S, Kang SB, Lim SB, Choi HS, Kim DW, Chang HJ, Kim DY, Jung KH, Kim TY, Kang GH, Chie EK, Kim SY, Sohn DK, Kim DH, Kim JS, Lee HS, Kim JH, Oh JH. Open versus laparoscopic surgery for mid-rectal or low-rectal cancer after neoadjuvant chemoradiotherapy (COREAN trial): survival outcomes of an open-label, non-inferiority, randomised controlled trial. Lancet Oncol. 2014 Jun;15(7):767-74. doi: 10.1016/S1470-2045(14)70205-0. Epub 2014 May 15. PubMed PMID: 24837215.

12. Oppedijk V, van der Gaast A, van Lanschot JJ, van Hagen P, van Os R, van Rij CM, van der Sangen MJ, Beukema JC, Rütten H, Spruit PH, Reinders JG, Richel DJ, van Berge Henegouwen MI, Hulshof MC. Patterns of recurrence after surgery alone versus preoperative chemoradiotherapy and surgery in the CROSS trials. J Clin Oncol. 2014 Feb 10;32(5):385-91. doi: 10.1200/JCO.2013.51.2186. Epub 2014 Jan 13. PubMed PMID: 24419108.

13. Ajani JA, Xiao L, Roth JA, Hofstetter WL, Walsh G, Komaki R, Liao Z, Rice DC,

Vaporciyan AA, Maru DM, Lee JH, Bhutani MS, Eid A, Yao JC, Phan AP, Halpin A, Suzuki A, Taketa T, Thall PF, Swisher SG. A phase II randomized trial of induction chemotherapy versus no induction chemotherapy followed by preoperative chemoradiation in patients with esophageal cancer. Ann Oncol. 2013 Nov;24(11):2844-9. doi: 10.1093/annonc/mdt339. Epub 2013 Aug 23. PubMed PMID: 23975663; PubMed Central PMCID: PMC3937600.

14. Nakamura K, Kato K, Igaki H, Ito Y, Mizusawa J, Ando N, Udagawa H, Tsubosa Y, Daiko H, Hironaka S, Fukuda H, Kitagawa Y; Japan Esophageal Oncology Group/Japan Clinical Oncology Group. Three-arm phase III trial comparing cisplatin plus 5-FU (CF) versus docetaxel, cisplatin plus 5-FU (DCF) versus radiotherapy with CF (CF-RT) as preoperative therapy for locally advanced esophageal cancer (JCOG1109, NExT study). Jpn J Clin Oncol. 2013 Jul;43(7):752-5. doi: 10.1093/jjco/hyt061. Epub 2013 Apr 26. PubMed PMID: 23625063.

15. van Heijl M, van Lanschot JJ, Koppert LB, van Berge Henegouwen MI, Muller K,

Steyerberg EW, van Dekken H, Wijnhoven BP, Tilanus HW, Richel DJ, Busch OR, Bartelsman JF, Koning CC, Offerhaus GJ, van der Gaast A. Neoadjuvant chemoradiation followed by surgery versus surgery alone for patients with adenocarcinoma or squamous cell carcinoma of the esophagus (CROSS). BMC Surg. 2008 Nov 26;8:21. doi: 10.1186/1471-2482-8-21. PubMed PMID: 19036143; PubMed Central PMCID: PMC2605735.

16. Kelsen DP, Winter KA, Gunderson LL, Mortimer J, Estes NC, Haller DG, Ajani JA, Kocha W, Minsky BD, Roth JA, Willett CG; Radiation Therapy Oncology Group; USA Intergroup. Long-term results of RTOG trial 8911 (USA Intergroup 113): a random assignment trial comparison of chemotherapy followed by surgery compared with surgery alone for esophageal cancer. J Clin Oncol. 2007 Aug 20;25(24):3719-25. PubMed PMID: 17704421.

17. Natsugoe S, Okumura H, Matsumoto M, Uchikado Y, Setoyama T, Yokomakura N, Ishigami S, Owaki T, Aikou T. Randomized controlled study on preoperative chemoradiotherapy followed by surgery versus surgery alone for esophageal squamous cell cancer in a single institution. Dis Esophagus. 2006;19(6):468-72. PubMed PMID: 17069590.

18. Burmeister BH, Smithers BM, Gebski V, Fitzgerald L, Simes RJ, Devitt P, Ackland S, Gotley DC, Joseph D, Millar J, North J, Walpole ET, Denham JW; Trans-Tasman Radiation Oncology Group; Australasian Gastro-Intestinal Trials Group. Surgery alone versus chemoradiotherapy followed by surgery for resectable cancer of the oesophagus: a randomised controlled phase III trial. Lancet Oncol. 2005 Sep;6(9):659-68. PubMed PMID: 16129366.

19. Fujita H, Sueyoshi S, Tanaka T, Tanaka Y, Sasahara H, Shirouzu K, Suzuki G,

Hayabuchi N, Inutsuka H. Prospective non-randomized trial comparing esophagectomy-followed-by-chemoradiotherapy versus chemoradiotherapy-followed-by-esophagectomy for T4 esophageal cancers. J Surg Oncol. 2005 Jun 15;90(4):209-19. PubMed PMID: 15906363.

20. Lee JL, Park SI, Kim SB, Jung HY, Lee GH, Kim JH, Song HY, Cho KJ, Kim WK, Lee JS, Kim SH, Min YI. A single institutional phase III trial of preoperative chemotherapy with hyperfractionation radiotherapy plus surgery versus surgery alone for resectable esophageal squamous cell carcinoma. Ann Oncol. 2004 Jun;15(6):947-54. PubMed PMID: 15151953

**5 studies without comparison:**

1. McDonnell CO, Harmey JH, Bouchier-Hayes DJ, Walsh TN. Effect of multimodality therapy on circulating vascular endothelial growth factor levels in patients with oesophageal cancer. Br J Surg. 2001 Aug;88(8):1105-9. PubMed PMID: 11488797.

2. Markar SR, Gronnier C, Pasquer A, Duhamel A, Behal H, Théreaux J, Gagnière J, Lebreton G, Brigand C, Meunier B, Collet D, Mariette C; FREGAT working group - FRENCH - AFC. Surgically treated oesophageal cancer developed in a radiated field: Impact on peri-operative and long-term outcomes. Eur J Cancer. 2017 Apr;75:179-189. doi: 10.1016/j.ejca.2016.12.036. Epub 2017 Feb 23. PubMed PMID: 28236769.

3. Hennessy TP. Cancer of the oesophagus. Postgrad Med J. 1996 Aug;72(850):458-63. Review. PubMed PMID: 8796207; PubMed Central PMCID: PMC2398530.

4. van Hagen P, Hulshof MC, van Lanschot JJ, Steyerberg EW, van Berge Henegouwen MI, Wijnhoven BP, Richel DJ, Nieuwenhuijzen GA, Hospers GA, Bonenkamp JJ, Cuesta MA, Blaisse RJ, Busch OR, ten Kate FJ, Creemers GJ, Punt CJ, Plukker JT, Verheul HM, Spillenaar Bilgen EJ, van Dekken H, van der Sangen MJ, Rozema T, Biermann K, Beukema JC, Piet AH, van Rij CM, Reinders JG, Tilanus HW, van der Gaast A; CROSS Group. Preoperative chemoradiotherapy for esophageal or junctional cancer. N Engl J Med. 2012 May 31;366(22):2074-84. doi: 10.1056/NEJMoa1112088. PubMed PMID: 22646630.

5. Rodriguez CP, Adelstein DJ, Rice TW, Rybicki LA, Videtic GM, Saxton JP, Murthy SC, Mason DP, Ives DI. A phase II study of perioperative concurrent chemotherapy, gefitinib, and hyperfractionated radiation followed by maintenance gefitinib in locoregionally advanced esophagus and gastroesophageal junction cancer. J Thorac Oncol. 2010 Feb;5(2):229-35. doi: 10.1097/JTO.0b013e3181c5e334. PubMed PMID: 20009775.

**3 non-randomized controlled trials:**

1. Spicer JD, Stiles BM, Sudarshan M, Correa AM, Ferri LE, Altorki NK, et al. Preoperative Chemoradiation Therapy Versus Chemotherapy in Patients Undergoing Modified En Bloc Esophagectomy for Locally Advanced Esophageal Adenocarcinoma: Is Radiotherapy Beneficial? Ann Thorac Surg. 2016;101(4):1262-9; discussion 969-70.

2. Luu TD, Gaur P, Force SD, Staley CA, Mansour KA, Miller JI, Jr., et al. Neoadjuvant chemoradiation versus chemotherapy for patients undergoing esophagectomy for esophageal cancer. Ann Thorac Surg. 2008;85(4):1217-23; discussion 23-4.

3. Morgan MA, Lewis WG, Crosby TD, Escofet X, Roberts SA, Brewster AE, et al. Prospective cohort comparison of neoadjuvant chemoradiotherapy versus chemotherapy in patients with oesophageal cancer. Br J Surg. 2007;94(12):1509-14.

1. **studies with overlapping study patients:**

1. Stahl M, Walz MK, Stuschke M, Lehmann N, Meyer HJ, Riera-Knorrenschild J,

Langer P, Engenhart-Cabillic R, Bitzer M, Königsrainer A, Budach W, Wilke H.

Phase III comparison of preoperative chemotherapy compared with chemoradiotherapy in patients with locally advanced adenocarcinoma of the esophagogastric junction. J Clin Oncol. 2009 Feb 20;27(6):851-6. doi: 10.1200/JCO.2008.17.0506. Epub 2009 Jan 12. PubMed PMID: 19139439.

2. Klevebro F, Johnsen G, Johnson E, Viste A, Myrnäs T, Szabo E, Jacobsen AB, Friesland S, Tsai JA, Persson S, Lindblad M, Lundell L, Nilsson M. Morbidity and mortality after surgery for cancer of the oesophagus and gastro-oesophageal junction: A randomized clinical trial of neoadjuvant chemotherapy vs. Neoadjuvant chemoradiation. Eur J Surg Oncol. 2015 Jul;41(7):920-6. doi: 10.1016/j.ejso.2015.03.226. Epub 2015 Apr 8. PubMed PMID: 25908010.

**1 study with insufficient data:**

1. Nakamura K, Kato K, Igaki H, Ito Y, Mizusawa J, Ando N, Udagawa H, Tsubosa Y, Daiko H, Hironaka S, Fukuda H, Kitagawa Y; Japan Esophageal Oncology Group/Japan Clinical Oncology Group. Three-arm phase III trial comparing cisplatin plus 5-FU (CF) versus docetaxel, cisplatin plus 5-FU (DCF) versus radiotherapy with CF (CF-RT) as preoperative therapy for locally advanced esophageal cancer (JCOG1109, NExT study). Jpn J Clin Oncol. 2013 Jul;43(7):752-5. doi: 10.1093/jjco/hyt061.Epub 2013 Apr 26. PubMed PMID: 23625063.
